# Anatomical study of the sphenopalatine foramen

**DOI:** 10.1016/S1808-8694(15)30829-6

**Published:** 2015-10-18

**Authors:** Adriana Bernardini Antunes Scanavine, João Adolfo Caldas Navarro, Silvia Regina Molinari de Carvalho Leitão Megale, Wilma Terezinha Anselmo-Lima

**Affiliations:** 1 Master’s degree in Otorhinolaryngology, professor.; 2 Livre-Docente certification, full professor.; 3 Master’s degree in Otorhinolaryngology, professor.; 4 Livre-Docente certification, associate professor.; Faculdade de Medicina de Ribeirão Preto, Universidade de São Paulo.

**Keywords:** epistaxis, ligation

## Abstract

Anatomical variations of the sphenopalatine foramen may correspond to alterations at the arterial nasal irrigation input, which is a relevant condition to treat severe epistaxis through ligation of the sphenopalatine artery. **Aim:** To study the sphenopalatine foramen in terms of its numeric variation and its location on the lateral nasal wall in relation to the bony ethmoidal crest of the palatine bone. **Materials and methods:** The anatomical studies were carried out in 54 hemifaces. **Results:** the sphenopalatine foramen presented the following numeric variation: single (87%, or 47 specimens), double (11,1%, or 6 specimens), and triple (1.9% or one specimen); it was located at the superior nasal meatus in 81.5%, or 44 specimens; 14.8% (8 specimens) between the middle and superior nasal meatus and in the middle nasal meatus in only one case (1.9%). **Conclusion:** We have been able to show a numeric variation of the SPF, its relation with the bony ethmoidal crest and its location in the superior meatus, middle meatus, and in both.

## INTRODUCTION

Nasal hemorrhage is one of the most common problems otorhinolaryngologists face in their specialty; severe cases may be characterized as a medical emergency.^1^ In these situations, therapeutic efficacy relates directly to an intervention as close as possible to the point of bleeding, the injured vessel. An essential step, therefore, is to identify that vessel; topographic diagnosis should be as precise as possible.^2^ The topographic diagnosis and surgical treatment of severe epistaxis require the surgeon to have, among other requirements, an adequate knowledge of the lateral nasal wall anatomy. Severe epistaxis is generally persistent and may cause hemodynamic instability and even death. The most frequent site of epistaxis is the postero-lateral wall of the nose, below the middle nasal turbinate, followed by the posterior septum.^2^, ^3^ Branches of the sphenopalatine artery irrigate this region; this vessel enters the nasal cavity by the sphenopalatine foramen (FEP).^4^ These branches are the posterior lateral and septal nasal arteries, according to the Anatomical Terminology (2001).^5^

The sphenopalatine foramen consists of a notch on the superior border of the palatine bone, between the orbital and sphenoid processes; the notch becomes a foramen at the point in which the palatine bone articulates with the sphenoid bone in the lateral nasal wall. It may be a complete orifice or traversed by one or more bony spiculae, suggesting more than one orifice.^6^ Some studies have shown that the shape of the sphenopalatine foramen may vary, becoming oval, square, triangular, piriform, and others;^7^, ^8^ its width ranges from four to seven millimeters and its height ranges from six to seven millimeters.^9^

Nikolic (1967)^8^ studied the numeric variation of the sphenopalatine foramen in 840 anatomical specimens, and found that 61.5% were single and 38.5% were multiple. Based on these numbers, recently published papers^10^, ^11^, ^12^ have suggested that there may be anatomical variants in the entrance point of the nasal arterial vessels (the branching pattern of the sphenopalatine artery, which in 95% of cases divides close to the sphenopalatine foramen, in the pterygopalatine fossa^10^); this may depend on the number of foramens, and may be the cause of failure in the treatment of severe epistaxis or failure in arterial ligature.^4^, ^12^, ^13^

According to some authors, the sphenopalatine foramen may be found in the superior nasal meatus in the nasal cavity.^6^, ^14^ However, if its position on the lateral nasal wall is established by the relation with the ethmoidal crest of the palatine bone, onto which it joins the posterior portion of the middle nasal turbinate, other studies locate it in the middle and/or superior nasal meatuses;^9^, ^13^ it is used as an anatomical landmark in endoscopic surgery.^15^

It is essential that surgeons possess ample knowledge of the anatomy, physiology, surgical techniques and complications,^16^ among other requirements, to undertake arterial ligature and other endoscopic procedures in the nasal cavity and avoid possible failures.^17^ Thus, the purpose of this study was do describe the number variation of the sphenopalatine foramen and its location relative to the bony crest of the middle nasal turbinate.

## MATERIAL AND METHOD

The Faculdade de Odontologia de Bauru, Universidade de Sao Paulo, exempted this study from requiring an approval protocol number from the Research Ethics Committee since it was conducted on anatomical specimens belonging to the Anatomy Department of Biological Sciences of that institution. We conducted anatomical studies of the nasal cavity in 54 female and male Caucasian and non-Caucasian adult half skulls. Nasofibroscopy was done initially in all half skulls to screen for anatomical variants or previous surgery. Half skulls consisted of median sagittally cut bone specimens. We aimed to identify the sphenopalatine foramen and its number variation by visually observing and photographing the half skulls. Anatomical observations to locate the sphenopalatine foramen relative to adjacent nasal cavity structures were done; this involved identifying the position of the bony crest of the middle nasal turbinate (COM) in relation to the foramen. The following criteria were used: the sphenopalatine foramen was considered as located in the superior nasal meatus (MS) when the posterior tip of the bony crest of the middle nasal turbinate pointed to the anterior and inferior border of the sphenopalatine foramen; it was considered as located in the middle nasal meatus (MM) when the bony crest of the middle nasal turbinate pointed to the anterior and superior border of the sphenopalatine foramen; and it was considered as located between the middle and superior nasal meatuses (MS-MM) when the bony crest of the middle nasal turbinate pointed to the median line of the sphenopalatine foramen. The parts that contained the bony crest of the superior nasal turbinate (COS) relative to the sphenopalatine foramen were also identified. A Nikon Coolpix 900 digital camera was used for photography. A 5-millimeter scale was placed next to each specimen as a size reference for each picture. The 54 color pictures were recorded on a CD-ROM, and are currently part of the author’s personal collection.

## RESULTS

**Figure 1:**
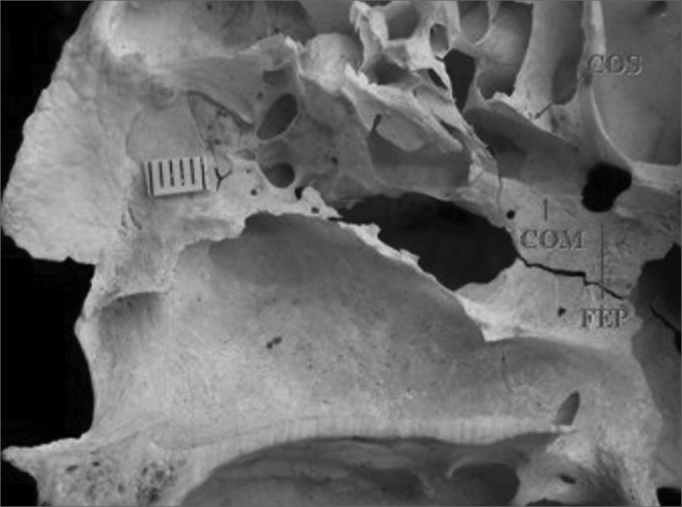
Single sphenopalatine foramen located in the superior nasal meatus in the right lateral osseous wall of the nasal cavity. Presence of bony crest on the superior nasal turbinate. Key: COM - bony crest on the middle nasal turbinate, COS - bony crest on the superior nasal turbinate, FEP - sphenopalatine foramen.

**Figure 2:**
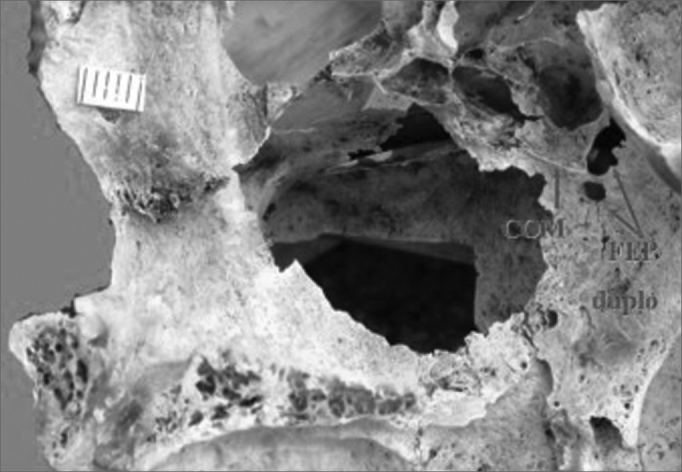
Double sphenopalatine foramen located in the superior and middle nasal meatuses in the right osseous wall of the nasal cavity. Key: COM - bony crest on the middle nasal turbinate, FEP - sphenopalatine foramen.

**Figure 3:**
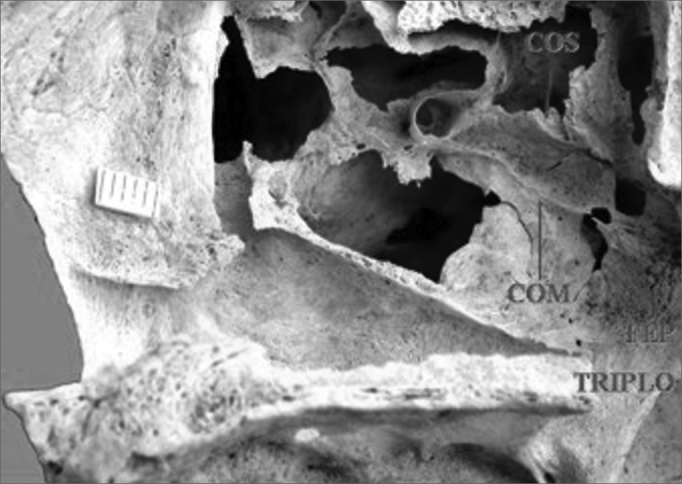
Triple sphenopalatine foramen located in the superior and middle nasal meatuses in the right osseous wall of the nasal cavity. Presence of bony crest on the superior nasal turbinate. Key: COS - bony crest superior, COM - bony crest on the middle nasal turbinate, FEP - sphenopalatine foramen.

In this study we found that 47 specimens contained single orifices (87%) (Fig. 1), six specimens had double orifices (11.1%) (Fig. 2), and one specimen presented a triple foramen (1.9%) (Fig. 3). Thus, seven specimens presented more than one orifice (13%).

**Figure 4:**
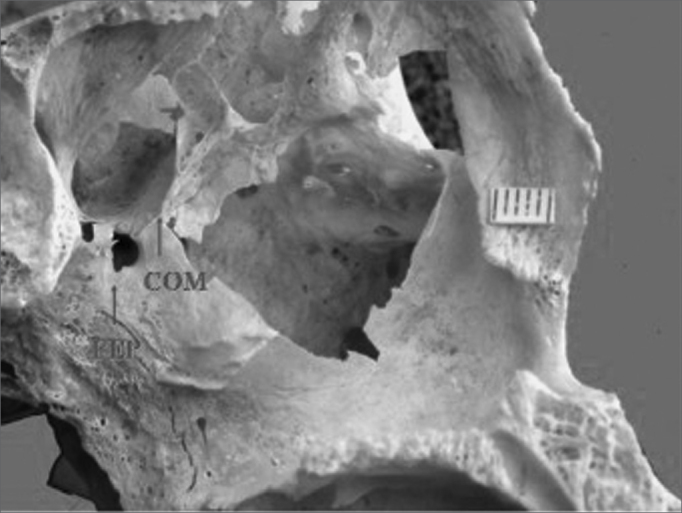
Single sphenopalatine foramen located in the middle nasal meatus in the left osseous wall of the nasal cavity. Key: COM - bony crest on the middle nasal turbinate, FEP - sphenopalatine foramen.

A study of the location of the sphenopalatine foramen on the lateral nasal wall relative to the bony crest middle nasal turbinate revealed that in 44 specimens the bony crest of the middle nasal turbinate pointed to the inferior border of the sphenopalatine foramen, placing it in the superior nasal meatus (MS) (Fig. 1). In eight specimens the bony crest of the middle nasal turbinate pointed to the middle of the sphenopalatine foramen, placing it between the middle nasal meatus (MM) and the superior nasal meatus (Figs. 2 and 3). In one specimen the bony crest of the middle nasal turbinate pointed to the superior border of the sphenopalatine foramen, placing it in the middle nasal meatus (Fig. 4). Identification of the site was not possible in one case.

**Figure 5:**
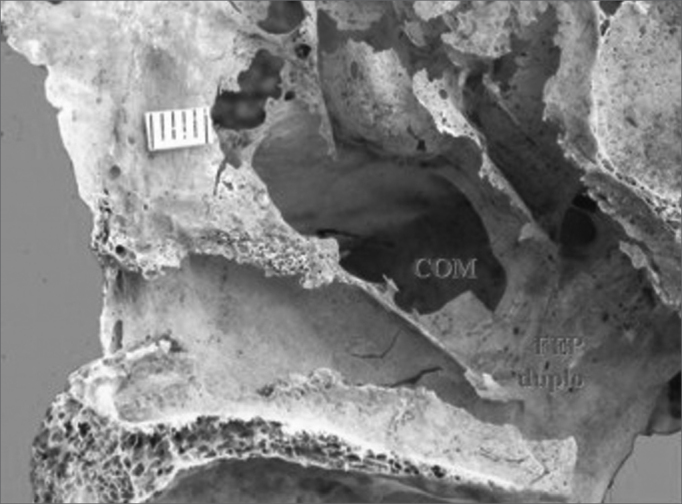
Double sphenopalatine foramen located in the superior nasal meatus in the right lateral osseous wall of the nasal cavity. Key: COM - bony crest on the middle nasal turbinate, FEP duplo - double sphenopalatine foramen.

We also found that in four specimens with double sphenopalatine foramens, these foramens were placed one above the other and had different sizes; the superior orifice was larger and the inferior orifice was the smallest. In these specimens, the bony crest of the middle nasal turbinate pointed to the sphenopalatine foramen in such a way as to indicate a superior and an inferior orifice (Fig. 2). In two other specimens, the bony crest of the middle nasal turbinate pointed to the inferior border of the sphenopalatine foramen, placing it in the superior meatus (Fig. 5).

In this study we were able to identify the presence of the bony crest of the superior nasal turbinate (COS) pointing to the sphenopalatine foramen in 30 specimens (55.6%) (Figs. 1 and 3).

## DISCUSSION

Our results support data in the literature about the number variation of the sphenopalatine foramen^7^, ^8^ by recording specimens with more than one foramen. Most of our samples had a single orifice (87%); the remaining had more than one orifice (13%). Among specimens with more than one orifice, 11.1% (six specimens) had double orifices. These percentages are similar to data by Bagatella (1986)^9^ who found 10% of double structured specimens, and data by Wareing and Padgham (1998)13 who reported 12% of double orifice cases in a 220-specimen sample. There was one specimen with a triple orifice in our sample (1.9%), a rare finding diverging from Nikolic (1967)8 who, in his vast sample, found 39 specimens (5.5%) with three orifices. It is difficult to explain the reason for such differences, but it may be presumed that these findings reveal different evolutionary paths of the foramen in human populations with diverse genomic features.

It is recognized that the number variation of the sphenopalatine foramen is probably the main element explaining the failure of surgery when ligating the branches of the sphenopalatine artery in the treatment of nasal bleeding.^9^, ^13^ This hypothesis is based on the fact that anatomical variants and number variation may occur at the entrance point of the main arterial branches of the septal artery and the posterior lateral nasal artery.^4^ Lee et al. (2002)^14^ recently demonstrated the presence of two to four branches of the sphenopalatine artery before the opening of the sphenopalatine foramen. It is thus reasonable to assume that, anatomically and surgically, the number variation of the sphenopalatine foramen corresponds to branch variants of the sphenopalatine artery; this may be, therefore, a complicating factor for surgery. Schwartzbauer et al. (2003)^12^ note that surgery may fail if dissection is not carried out up to the posterior portion of the sphenopalatine foramen, due to the presence of arterial branches exiting through accessory foramens.

Given the importance of identifying the bony ethmoidal crest of the palatine bone, onto which the middle nasal turbinate is linked, and which is an anatomical and surgical landmark for locating the sphenopalatine foramen in an endonasal access, that structure was also observed. Our results revealed that in 44 specimens (81.5%) the distal tip of the bony crest of the middle nasal turbinate pointed towards the inferior margin of the sphenopalatine foramen, locating it in the superior nasal meatus; the sphenopalatine foramen was located between the superior and middle nasal meatuses in eight specimens (14%); the foramen was fully located in the middle meatus in only one specimen (1.9%). These results disagree with those that place the sphenopalatine foramen only in the superior nasal meatus,^6^, ^14^, ^15^ but support that data by Bagatella (1986)^9^ that 85% of sphenopalatine foramens are located in the superior nasal meatus, 5% in the middle nasal meatus, and 10% between both meatuses. Wareing and Padgham (1998)^13^ also reported that the sphenopalatine foramen was located in the superior nasal meatus in 35% of cases, and between the superior and middle nasal meatuses in 65% of cases. These authors looked at anatomical site variations in bone specimens of the sphenopalatine foramen in the superior and middle nasal meatuses. They suggested that a mucoperiostal flap be made above and below the middle nasal turbinate, about one centimeter from its posterior tip, in surgery for ligating or cauterizing branches of the sphenopalatine artery, to avoid missing any of the foramens. Our data support these authors, and suggest the same surgical approach.

Aside from the comments about the bony crest of the middle nasal turbinate, we also noted that in 30 specimens (55.6%), the bony crest of the superior nasal turbinate pointing towards the superior border of the sphenopalatine foramen. This finding has also been often reported.^14^, ^15^, ^18^ The role of this structure as a landmark for surgery remains unclear, and has not been discussed by other authors.

Our study showed a single sphenopalatine foramen in most of the study specimens, although there were double and tripe foramens. We noted the number variation of the sphenopalatine foramen, and consequently of the branches of the sphenopalatine artery, supporting the surgical treatment of severe epistaxis with fewer failures.
